# Serious Bacterial Infections Acquired During Treatment of Patients Given a Diagnosis of Chronic Lyme Disease — United States

**DOI:** 10.15585/mmwr.mm6623a3

**Published:** 2017-06-16

**Authors:** Natalie S. Marzec, Christina Nelson, Paul Ravi Waldron, Brian G. Blackburn, Syed Hosain, Tara Greenhow, Gary M. Green, Catherine Lomen-Hoerth, Marjorie Golden, Paul S. Mead

**Affiliations:** ^1^Preventive Medicine Residency, University of Colorado, Aurora, Colorado; ^2^Division of Vector-Borne Diseases, National Center for Emerging and Zoonotic Infectious Diseases, CDC; ^3^Kaiser Permanente, San Jose, California; ^4^Division of Infectious Diseases and Geographic Medicine, Stanford University School of Medicine, Stanford, California; ^5^LifeBridge Health, Westminster, Maryland; ^6^Kaiser Permanente, Santa Rosa, California; ^7^University of California, San Francisco, California; ^8^Yale University School of Medicine, New Haven, Connecticut.

The term “chronic Lyme disease” is used by some health care providers as a diagnosis for various constitutional, musculoskeletal, and neuropsychiatric symptoms (*1*,*2*). Patients with a diagnosis of chronic Lyme disease have been provided a wide range of medications as treatment, including long courses of intravenous (IV) antibiotics (*3*,*4*). Studies have not shown that such treatments lead to substantial long-term improvement for patients, and they can be harmful (*1*,*5*). This report describes cases of septic shock, osteomyelitis, *Clostridium difficile* colitis, and paraspinal abscess resulting from treatments for chronic Lyme disease. Patients, clinicians, and public health practitioners should be aware that treatments for chronic Lyme disease can carry serious risks.

Lyme disease is a well-known condition caused by infection with the spirochete *Borrelia burgdorferi* sensu lato. Features of early infection include erythema migrans (an erythematous skin lesion with a bull’s-eye or homogeneous appearance), fever, headache, and fatigue. If left untreated, the spirochete can disseminate throughout the body to cause meningitis, carditis, neuropathy, or arthritis (*5*,*6*). The recommended treatment for Lyme disease is generally a 2–4-week course of antibiotics (*5*). 

Chronic Lyme disease, on the other hand, is a diagnosis that some health care providers use to describe patients with a variety of conditions such as fatigue, generalized pain, and neurologic disorders. Many of these patients have experienced significant debilitation from their symptoms and have not found relief after consultation with conventional medical practitioners. As a result, some seek treatment from practitioners who might identify themselves as Lyme disease specialists (“Lyme literate” doctors) or from complementary and alternative medicine clinics, where they receive a diagnosis of chronic Lyme disease (*3*,*7*). 

A diagnosis of chronic Lyme disease might be based solely on clinical judgment and without laboratory evidence of *B. burgdorferi* infection, objective signs of infection, or a history of possible tick exposure in an area with endemic Lyme disease (*1*,*7*). There is a belief among persons who support the diagnosis and treatment of chronic Lyme disease that *B. burgdorferi* can cause disabling symptoms even when standard testing is negative, despite evidence that the recommended two-tiered serologic testing is actually more sensitive the longer *B. burgdorferi* infection has been present (*6*). Some practitioners use tests or testing criteria that have not been validated for the diagnosis of Lyme disease (*1*). A significant concern is that after the diagnosis of chronic Lyme disease is made, the actual cause of a patient’s symptoms might remain undiagnosed and untreated (*3*,*8*).

Patients given a diagnosis of chronic Lyme disease have been prescribed various treatments for which there is often no evidence of effectiveness, including extended courses of antibiotics (lasting months to years), IV infusions of hydrogen peroxide, immunoglobulin therapy, hyperbaric oxygen therapy, electromagnetic frequency treatments, garlic supplements, colloidal silver, and stem cell transplants (*1*,*3*). At least five randomized, placebo-controlled studies have shown that prolonged courses of IV antibiotics in particular do not substantially improve long-term outcome for patients with a diagnosis of chronic Lyme disease and can result in serious harm, including death (*1*,*5*,*9*).*

Clinicians and state health departments periodically contact CDC concerning patients who have acquired serious bacterial infections during treatments for chronic Lyme disease. Five illustrative cases described to CDC over the past several years are presented.

## Patient A

A woman in her late 30s with fatigue and joint pain received a diagnosis of chronic Lyme disease, babesiosis, and *Bartonella* infection by a local physician. Despite multiple courses of oral antibiotics, her symptoms worsened, and a peripherally inserted central catheter (PICC) was placed for initiation of IV antibiotic treatment. After 3 weeks of treatment with IV ceftriaxone and cefotaxime, the patient’s joint pain continued, and she developed fever and rash. She became hypotensive and tachycardic and was hospitalized in an intensive care unit, where she was treated with broad spectrum IV antibiotics and required mechanical ventilation and vasopressors. Despite maximal medical support, she continued to worsen and eventually died. The patient’s death was attributed to septic shock related to central venous catheter–associated bacteremia.

## Patient B

An adolescent girl sought medical advice regarding years of muscle and joint pain, backaches, headaches, and lethargy. She had received a diagnosis of chronic fatigue syndrome, but sought a second opinion from an alternative medicine clinic and was told she had chronic Lyme disease. The patient was treated with oral antibiotics, including rifampin, trimethoprim-sulfamethoxazole, and doxycycline, for 3 months; these were discontinued because of abnormal liver enzyme test results Three months later, a PICC was placed to administer IV antibiotics, including ceftriaxone. After receiving both IV and oral antibiotic therapy for 5 months without improvement, the antibiotics were discontinued, but the PICC was not removed.

One week after antibiotics had been discontinued, the patient developed pallor, chills, and fever to 102.9°F (39.4°C); after consultation with the alternative medicine clinic, she was given another dose of ceftriaxone through the PICC. Later that day she was evaluated in an emergency department with fever to 105.3°F (40.7°C), hypotension, and tachycardia consistent with septic shock. Blood and PICC tip cultures grew *Acinetobacter* spp. She was hospitalized in an intensive care unit and required vasopressors as well as broad-spectrum antibiotics to treat the infection. The PICC was removed, and the patient was eventually discharged after several weeks of hospitalization.

## Patient C

A woman in her late 40s received multiple arthropod bites and subsequently developed a flu-like illness with pain in her arms, legs, and back. One year after her symptoms began, she received a diagnosis of Lyme disease using the recommended two-tiered serologic test (positive enzyme immunoassay test result followed by positive immunoglobulin G Western immunoblot). She was treated with two 4-week courses of oral doxycycline.

The patient developed fatigue, cognitive difficulties, and poor exercise tolerance, and 2 years after her initial diagnosis she received a diagnosis of chronic Lyme disease based on the results of unvalidated tests. She was treated with intramuscular penicillin for approximately 5 weeks without improvement, then IV ceftriaxone for 4 months, followed by IV azithromycin for 6 months administered via a tunneled IV catheter.

One year later, she received additional IV ceftriaxone via a new IV catheter, plus oral doxycycline, tinidazole (an antiparasitic medication), and azithromycin for approximately 4 weeks. The patient developed back pain, shortness of breath, and malaise, and was hospitalized. The catheter was removed, and blood and catheter tip cultures yielded *Pseudomonas aeruginosa*. She was treated with aztreonam for 4 weeks; however, her back pain worsened, and she was readmitted to the hospital. A computed tomography scan indicated destruction of both the 9th and 10th thoracic vertebrae, and magnetic resonance imaging of her spine confirmed osteodiscitis. A bone biopsy and culture grew *P. aeruginosa* with the same antibiotic susceptibility profile as her previously diagnosed bacteremia. She was treated for osteodiscitis, and her back pain eventually improved.

## Patient D

A woman in her 50s developed progressive weakness, swelling, and tingling in her extremities and received a tentative diagnosis of chronic inflammatory demyelinating polyneuropathy. Despite various treatments over a 5-year period, her symptoms did not substantially improve, and a diagnosis of amyotrophic lateral sclerosis was made.

The patient was subsequently evaluated by another physician and was told she had chronic Lyme disease, babesiosis, and Rocky Mountain spotted fever. Initial treatment with herbs and homeopathic remedies had no effect. She was treated with IV ceftriaxone and oral trimethoprim-sulfamethoxazole, acyclovir, fluconazole, and tinidazole. After 7 months of intensive antimicrobial treatment, her pain improved, but the weakness worsened. She discontinued treatment after developing *C. difficile* colitis that caused severe abdominal cramps and diarrhea. The *C. difficile* infection became intractable, and her symptoms persisted for over 2 years, requiring prolonged treatment. The patient subsequently died from complications of amyotrophic lateral sclerosis.

## Patient E

A woman in her 60s with autoimmune neutropenia, mixed connective tissue disease, and degenerative arthritis received a diagnosis of chronic Lyme disease neuropathy, for which she received IV immunoglobulin every 3 weeks via a tunneled venous catheter with an implanted subcutaneous port. After undergoing treatments for >10 years, she developed fevers and neck pain and was hospitalized; the catheter was removed, and blood and catheter tip cultures yielded methicillin-sensitive *Staphylococcus aureus*. She was treated with IV antibiotics via a newly placed PICC. Although the patient was advised to have the PICC removed once the antibiotic course finished, she chose to keep it for further IV immunoglobulin therapy.

Two months later, she was readmitted for recurrent fevers. The PICC was removed, and cultures of the tip grew coagulase-negative *Staphylococcus*; blood cultures were negative. She was treated with IV antibiotics and discharged.

The patient subsequently received a new implanted subcutaneous venous catheter and restarted IV immunoglobulin therapy, after which she was readmitted for fever and back pain. Blood cultures were positive for methicillin-sensitive *S. aureus*, and magnetic resonance imaging indicated inflammation of the lumbar facet joints, epidural space, and paraspinal muscles, consistent with infection. Despite appropriate antibiotic treatment, her back pain worsened, and she required surgical drainage of a paraspinal abscess.

## Discussion

Antibiotics and immunoglobulin therapies are effective and necessary treatments for many conditions; however, unnecessary antibiotic and immunoglobulin use provides no benefit to patients while putting them at risk for adverse events. When used for extended periods, the risks associated with these treatments increase, so it is important that they be used appropriately.

These cases highlight the severity and scope of adverse effects that can be caused by the use of unproven treatments for chronic Lyme disease. In addition to the dangers associated with inappropriate antibiotic use, such as selection of antibiotic-resistant bacteria, these treatments can lead to injuries related to unnecessary procedures, bacteremia and resulting metastatic infection, venous thromboses, and missed opportunities to diagnose and treat the actual underlying cause of the patient’s symptoms (*8*,*10*).^†^^,^^§^ Patients and their health care providers need to be aware of the risks associated with treatments for chronic Lyme disease.

The number of persons who undergo treatments for chronic Lyme disease is unknown, as is the number of complications that result from such treatments. Systematic investigations would be useful to understand the scope and consequences of adverse effects resulting from treatment of persons with a diagnosis of chronic Lyme disease. Data sources to consider include clinician surveys, administrative claims databases, or implementation of state or local reporting systems for adverse outcomes related to these treatments.

**Summary**

**What is already known about this topic?**

“Chronic Lyme disease” is a nonspecific diagnosis without a consistent definition that has been given to patients with various symptoms. Treatments offered for chronic Lyme disease, such as prolonged antibiotic or immunoglobulin therapy, lack data supporting effectiveness and are not recommended.

**What is added by this report?**

Clinicians, health departments, and patients have contacted CDC with reports of serious bacterial infections resulting from treatment of persons who have received a diagnosis of chronic Lyme disease. Five of these cases are described to illustrate complications resulting from unproven treatments, including septic shock, *Clostridium difficile* colitis, osteodiscitis, abscess, and death.

**What are the implications for public health practice?**

Clinicians, public health practitioners, and patients should be aware that treatments for chronic Lyme disease lack proof of effectiveness and can result in serious complications. Systematic investigation into the scope and effects of these complications, including the rate and extent of infections and the pathogens associated with these infections, would be helpful to inform clinical practice and fully characterize the risks associated with treatments for chronic Lyme disease.

## Footnotes

* https://www.cdc.gov/lyme/treatment/prolonged/index.html.

† https://www.cdc.gov/features/getsmart/.

§ https://www.cdc.gov/hai/bsi/bsi.html.
